# The role of sigmoidoscopy in colorectal cancer screening – public preferences and predicted uptake

**DOI:** 10.1186/s12889-026-30227-w

**Published:** 2026-11-13

**Authors:** Melanie Brinkmann, Kathrin Krüger, Melissa Hemmerling, Jona Theodor Stahmeyer, Bernt-Peter Robra, Christian Krauth, Maren Dreier

**Affiliations:** 1 Hannover Medical School, Institute for General Practice and Palliative Care, Carl-Neuberg Str. 1, Hannover, D-30625 Germany; 2 Hannover Medical School, Institute for Epidemiology, Social Medicine and Health Systems Research, Hannover, Germany; 3 Center for Health Economic Research Hannover, Hannover, Germany; 4 Health Services Research Unit, AOK Niedersachsen, Hannover, Germany; 5 Institute of Social Medicine and Health Systems Research, Otto-von-Guericke University Magdeburg, Magdeburg, Germany

**Keywords:** Colorectal cancer, Early diagnosis, Preferences, Colonoscopy, Sigmoidoscopy, Discrete choice experiment

## Abstract

**Background:**

Participation in cancer screening is crucial to maximise its benefit. In colorectal cancer (CRC) screening, stool tests or colonoscopies are commonly offered worldwide screening tests. In the German CRC screening programme, the insured can primarily choose between stool test and colonoscopy. However, sigmoidoscopy is also an evidence-based procedure to reduce CRC mortality and even prevent CRC. Based on individual preferences, we aim to estimate the uptake for either colonoscopy, sigmoidoscopy, or stool test in CRC screening.

**Methods:**

In 2020, statutory insured individuals aged 50, 55, and 60 in Germany, Lower Saxony, were invited to participate in a discrete choice experiment (DCE). Each participant received 8 choice tasks, each of which presented two unlabelled (procedure A and B) screening alternatives defined by 6 attributes (CRC incidence and mortality, complications, preprocedural bowel preparation, need for transportation home, colonoscopy following a positive test result). Each attribute had 3 levels. To calculate the indirect utility and probability of uptake of stool testing, sigmoidoscopy, colonoscopy, and ‘no screening’, the preference weights of the attribute levels used to define the 4 alternatives were summed. Estimates were compared between standard screening (stool test or colonoscopy) and extended screening (+ sigmoidoscopy). External validity of the DCE was checked by comparing predicted and intended (2 items in the questionnaire) participation.

**Results:**

From *n* = 666 valid respondents, a greater proportion of women (22.5%; 95%CI 16.2–28.9%) than men (13.4%; 95%CI 9.6–17.2%) preferred sigmoidoscopy in an extended screening scenario. Compared with standard screening, the predicted uptake for colonoscopy and the stool test as well as for ‘no screening’ was slightly lower when sigmoidoscopy was offered as an additional option. The extended screening could yield a slightly higher non-significant total participation in CRC screening in both women (+ 5.8%; 95%CI -0.4-12.0%) and men (+ 3.1; 95%CI -3.4-9.6%) than standard screening. Intended and preferred uptake did not differ substantially.

**Conclusions:**

A relevant proportion of the eligible population, more women than men, appears to prefer sigmoidoscopy for CRC screening when given the option. This additional option tends to increase overall predicted participation in CRC screening. Future studies are needed to evaluate the acceptance and feasibility of the additionally offered sigmoidoscopy in today’s CRC screening.

**Trial registration:**

DRKS00019010, 2019-11-18

**Supplementary Information:**

The online version contains supplementary material available at https://doi.org/10.1186/s12889-026-30227-w.

## Background

When we started the SIGMO (Sigmoidoscopy as an evidence based colorectal cancer screening test – a possible option? ) study in 2019, we wanted to find out whether insurees living in Germany would prefer the sigmoidoscopy for colorectal cancer (CRC) screening when offered [1]. Would it make sense to offer an additional screening test in the German health system, where people already can choose between the stool test or colonoscopy? It should be noted that sigmoidoscopy has been shown in four randomised controlled trials (RCTs) to reduce CRC mortality and to prevent CRC [2–5], and that it involves less effort than colonoscopy which is considered the gold standard in CRC screening. Specifically, sigmoidoscopy typically involves only a preparatory enema and is usually performed without sedation, while colonoscopy requires full bowel cleansing with fasting and drinking a large amount of laxative solution and is almost always performed with sedation. Since sigmoidoscopy usually does not require sedation, there are fewer side-effects, and no other person is needed to go home [6, 7]. Compared to colonoscopy, sigmoidoscopy causes fewer complications such as bleeding and bowel perforation [8, 9]. In sum, three highly evidence-based options are available for CRC screening: stool blood test, sigmoidoscopy, and colonoscopy. Why not offer all of them?

A closer look at the current evidence supporting the effectiveness of CRC screening shows that it varies across screening modalities [4]. Sigmoidoscopy has the most robust randomised evidence, demonstrating relative reductions in CRC incidence of approximately 17–30% and CRC mortality of 20–31% in intention-to-screen (ITS) analyses [2–4]. For colonoscopy, the randomised NordICC trial demonstrated a 19% rela​tive reduction in CRC incidence after 13 years, while per-protocol (PP) analyses suggested a 45% risk reduction [9, 10]. A statistically significant reduction in CRC​ mortality was not observed after 13 years in the ITS​ analysis; however, PP​ analyses indicated a non-significant 30% reduction in mortality [10]. In conclusion, despite its ability to visualize the entire colorectum, colonoscopy has not yet demonstrated clear superiority over sigmoidoscopy in randomised trials. This may partly be explained by the relatively low uptake of colonoscopy (42% participation rate) in NordICC, together with the low observed CRC mortality in the control group, which reduces the statistical power to detect additional mortality benefits [10, 11]. Strong evidence also supports the use of the faecal immunochemical test (FIT). Repeated FIT-based screening programs have been shown to reduce CRC​ mortality and achieved comparable 10-year outcomes to a one-time colonoscopy in the COLONPREV trial [12]. Overall, all established screening strategies reduce the burden of CRC, and at the population level, screening participation appears to be a key determinant of effectiveness.

In Germany, participation in CRC screening is still moderate, though since July 2019 we have an organised and quality-assured CRC screening programme for people with average CRC risk including a written invitation to participate and an information leaflet every five years starting at the age of 50 [13]. The 10-year uptake rate of screening colonoscopies among those with statutory health insurance is 27.2% for women and 26.3% for men [14]. Annual participation among women/men aged 50 to 54 in stool tests (2024) was 13.1%/3.8%, and biennial uptake among women/men aged 55 years and older (2023-24) was 10,1%/7,8% [7]. However, stool-testing outside the programme cannot be excluded. Offering another test option could increase participation, because some people may prefer the additional option of sigmoidoscopy for CRC screening [15]. As each test incorporates different benefits, harms, and efforts, offering a choice of screening tests may better align decisions with individual preferences and may be associated with fewer decisional conflicts and a higher rate of test completion, provided that appropriate evidence-based information is made available to support informed decision-making [15–19].

So far, little is known about participation in sigmoidoscopy when offered as an additional option in a multimodal CRC screening program [20]. To learn more about the preferences of the screening-eligible population, the SIGMO study used a discrete choice experiment (DCE) [1, 21, 22]. The results revealed heterogenous preferences among German insurees [23]. Some preferred most the benefit through preventing CRC incidence or death from CRC, while others valued comprehensive bowel preparation most. Based on these revealed preferences, we will now take a further step by estimating the predicted and intended screening uptake for either colonoscopy, sigmoidoscopy, or stool test.

## Methods

The SIGMO study evaluates whether sigmoidoscopy should be additionally offered for CRC screening in Germany [1]. Part of the study was to elicit the stated preferences of screening eligible individuals for different screening tests using a DCE. Eligible for inclusion were 50-year-old men, 55-year-old women (representing the sex-specific age of eligibility for the first screening colonoscopy until 2025), and 60-year-old women and men with statutory health insurance through the AOK Lower Saxony who were invited to participate in a written, postal questionnaire between June and October 2020. Ethical approval was obtained from the Ethics Committee of Hannover Medical School (reference number 8671_BO_K_2019), and the SIGMO study was registered at the German Clinical Trials Register (DRKS00019010). The conduct and reporting of the DCE was based on a checklist of the International Society for Pharmacoeconomics and Outcomes Research (ISPOR) Good Research Practices for Conjoint Analysis Task Force [24] and on the DIRECT (Discrete choice experiment Reporting Checklist) checklist [25].

### A brief overview of the discrete choice experiment

A detailed description of the identification and selection of the attributes and attribute levels, the construction of the choice tasks, the experimental and questionnaire design, the sampling and data collection processes, as well as the results of the DCE based on a conditional logit model, a random-parameters logit, and a latent-class-model to account for preference heterogeneity across respondents have been published elsewhere [23, 26, 27]. In short, stated preferences were elicited by unlabelled, two-alternative choice tasks without an opt-out alternative [28–30]. A fractional factorial design with 32 choice sets was divided into four blocks of eight choice tasks each. To evaluate the internal validity of the choice data, a within-set dominated pair was additionally included [31], so that each participant answered nine choice tasks in total. The unlabelled alternatives were defined by six attributes with three levels each: (1) CRC incidence (levels 3/1,000; 8/1,000; 10/1,000), (2) CRC mortality (1/1,000; 2/1,000; 3/1,000), (3) complications (0/1,000; 1/1,000; 2/1,000), (4) preprocedural bowel preparation (none; enema before examination; drinking 2–4 ​l and avoiding solid food), (5) need for transportation home (never; occasionally; almost always), and (6) colonoscopy following a positive test result (0/1,000; 340/1,000; 440/1,000). Within each choice set, individuals were asked to choose their preferred alternative (Table 1).

**Table 1:** Example of a choice set used in the discrete choice experiment

Which of the procedures would you prefer?		
Characteristics	Procedure A	Procedure B
Develop bowel cancer	8 out of 1,000	10 out of 1,000
Die from bowel cancer	2 out of 1,000	1 out of 1,000
Complications	1 out of 1,000	2 out of 1,000
Preparations	None	Enema before examination
Support needed to go home	Occasionally	Almost always
Follow-up after a positive test result	0 out of 1,000	340 out of 1,000
I prefer …	☐	☐

Of 5,000 individuals invited by mail, 4,945 were delivered; 1,282 questionnaires were returned (1,282/4,945 = 25,9% response rate); and 1,142 were included in the previously published analyses of the elicited choice data regarding the decision relevance of the attributes [23]. To account for an unexpectedly high proportion of participants (476/1,142 = 41.7%) choosing the dominated alternative (all attribute levels were inferior to those of the competing alternative) in the internal validity choice set, which is considered an irrational decision, the models used in the previous analyses were stratified by responses to the within-set dominated pair [31, 32]. As the reasons for choosing the dominated alternative in the internal validity choice set remained unclear, only respondents who had chosen the dominant alternative (all attribute levels were superior to those of the competing alternative) will be considered for the current statistical analyses (*n* = 666).

### Statistical analyses

Analyses aimed to estimate the screening uptake for either colonoscopy, sigmoidoscopy, or stool test based on the revealed preferences. A mixed logit model was used, assuming preference heterogeneity within the sample. The four quantitative attributes (CRC incidence, CRC mortality, complications, and follow-up) were included as continuous variables, whereas effects-coding was used for the two qualitative ones (preprocedural bowel preparation, need for transportation home). All analyses were stratified for sex. Model estimation and calculation of indirect utility and probability of uptake were conducted in STATA (StataCorp LLC, TX; Version 15.1).

#### Indirect utility and probability of uptake of screening tests

The indirect utility V of a screening test is the sum of the preference weights of the attribute levels used to define the respective alternative [21]. The probability P that individual n chooses screening test i from all possible procedures j equals

$$\:{P}_{in}=\frac{\mathrm{e}\mathrm{x}\mathrm{p}\left(\mu\:{V}_{in}\right)}{\sum\:\mathrm{e}\mathrm{x}\mathrm{p}\left(\mu\:{V}_{jn}\right)}$$

where V represents the previously calculated indirect utility of the respective alternative. The screening tests were defined by the attribute levels (see Additional File 1, Table 1). ‘No screening’ was also defined by attribute levels, because no opt-out alternative was included in the choice tasks. To evaluate the effect of the additional offer of sigmoidoscopy for CRC screening, the probability of uptake of screening tests was calculated for two different strategies: (a) standard screening comprising a choice between FIT, colonoscopy, or ‘no screening’, and (b) extended screening including a choice from FIT, colonoscopy, sigmoidoscopy, or ‘no screening’.

**Fig. 1:**
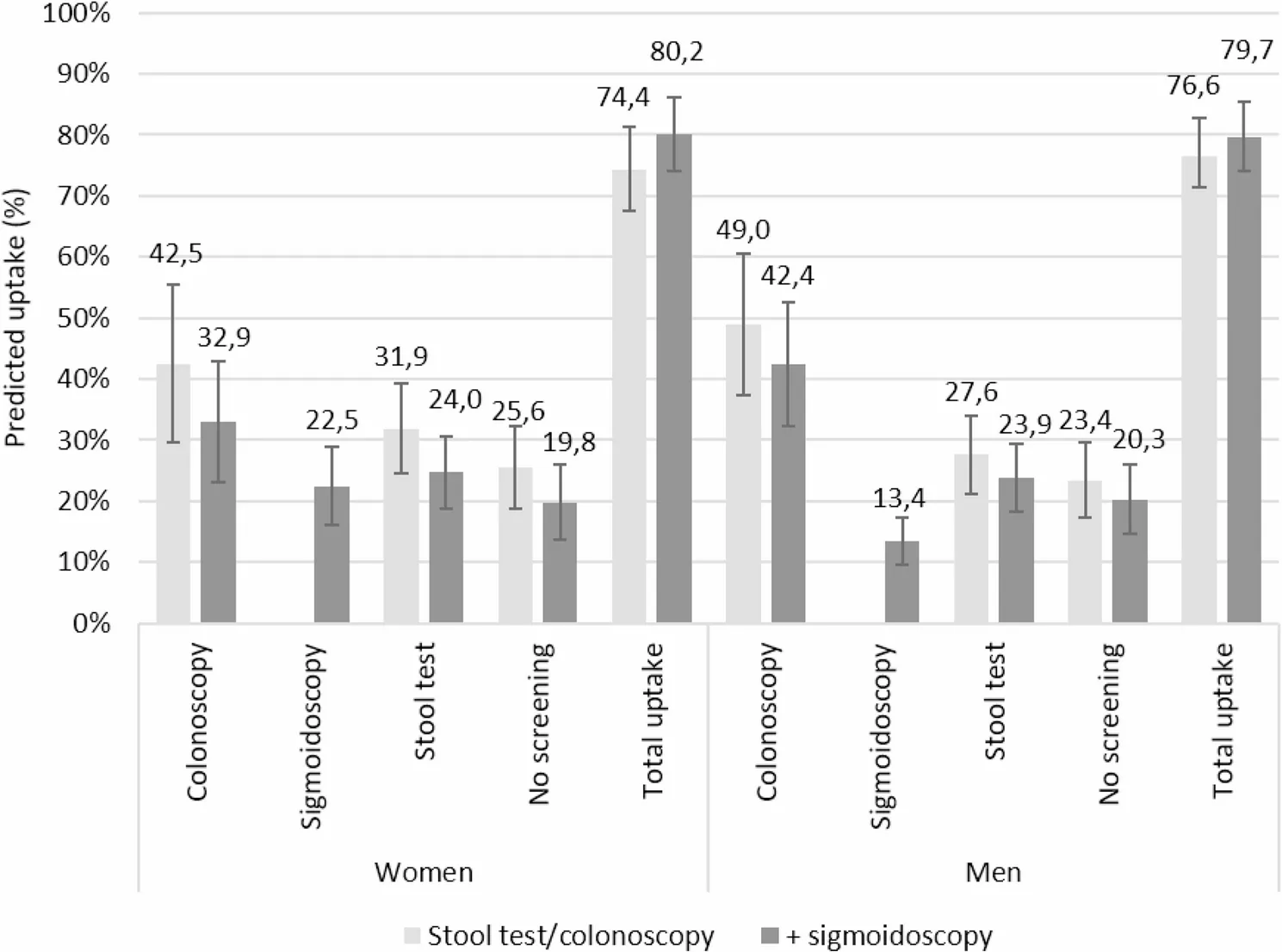
Predicted uptake (%) for colorectal cancer screening tests in standard screening (stool test​/colonoscopy) and screening extended by sigmoidoscopy (+ sigmoidoscopy) among women and men

**Fig. 2:**
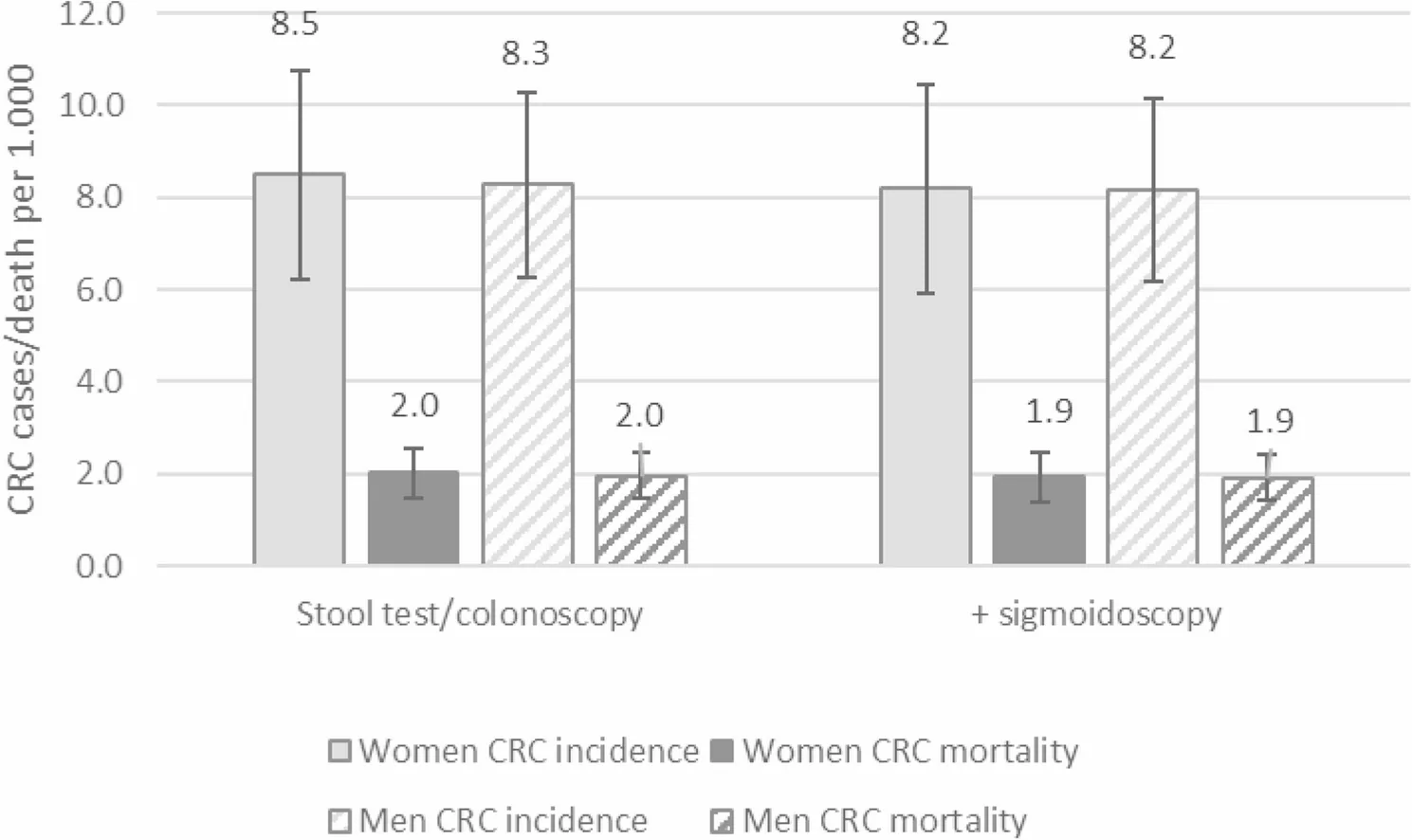
Estimated effects on CRC incidence and mortality of preference-based total uptakes of standard screening (stool test/colonoscopy) and screening extended by sigmoidoscopy (+ sigmoidoscopy) among women and men

For the definition of the screening tests, effectiveness level values (CRC incidence and mortality; see Additional File 1, Table 1) were modified for several reasons. The effectiveness level values originally selected for the DCE were based on the invitation and decision aid within the organised CRC screening programme in Germany to ensure consistency between the attribute levels used in the DCE and the information leaflet (version 2019) that respondents had received a few weeks earlier [33]. As a result, the effectiveness level values representing the stool test were based on RCTs​ on the effectiveness of gFOBT (Guaiac-based faecal occult blood test), and for colonoscopy on a meta-analysis of observational studies. To reduce the cognitive burden for participants, absolute risks of developing and dying from CRC were also presented as rounded values in the DCE. For the present analysis, the effectiveness level values of colonoscopy and sigmoidoscopy were based on results from PP​ analyses of RCTs [2, 9], while results from observational studies, and ITS​ analyses of RCTs were used for sensitivity analyses as presented in the supplement (Additional File 1, Table 2; Figs. 1 and 2). The study team considered the PP analysis to be most relevant for individual decision-making because it estimates the effect among individuals who actually undergo screening [34]. In contrast, ITS ​analyses evaluate the effectiveness of a screening offer at the population level and are influenced by non-participation and other factors unrelated to the efficacy of the screening procedure itself [35]. In addition, data on what happens in people who actually take part in CRC screening was needed, which is provided rather in PP analyses than in ITS analysis. ITS analysis underestimates the true effects due to incomplete adherence of the screening group and contamination in the control group; the effectiveness results of observational studies are much higher. For FIT, results from RCTs are not yet available. Thus, the ratio in effectiveness between FIT and sigmoidoscopy from observational studies was applied.

#### Effects on CRC incidence and mortality

To examine the effect of additionally offering sigmoidoscopy for CRC incidence and mortality, the predicted uptake rates for screening tests in standard and extended screening were multiplied by the effectiveness level values used and then subtracted from each other.

#### Intended screening uptake

The questionnaire included two items to collect the intention to participate: (1) Are you planning to participate in bowel cancer screening? (yes/no), and if yes, (2) what bowel cancer screening procedure do you plan to participate in? (stool test, colonoscopy, undecided). Predicted screening uptake (yes/no) based on previously calculated probabilities of uptake in standard screening and intended uptake was compared between women and men using Chi² tests in SPSS (IBM Corp, Armonk, NY; Version 29.0).

## Results

The analysis was based on *n* = 666 respondents. Of these, *n* = 624 provided valid responses to the questions on intended uptake. Table 2 describes the characteristics of the study population. The sample included slightly more women than men, more than a third had a low educational level, nearly 15% reported a migration background, and 40% of them were eligible to participate in screening colonoscopy for the first time. Almost 80% had had a stool test before and more than half of them a prior colonoscopy. Three quarters intended to participate in CRC screening.

**Table 2:** Self-reported characteristics of the study population

Characteristics	*n* = 666	(%)
Sex	(*n* = 660)	
Female	346	(52.4)
Male	314	(47.6)
Age, years	(*n* = 663)	
50	121	(18.3)
55	138	(20.8)
60	404	(60.9)
Sex, age (years)^a^	(*n* = 658)	
Female, 55	138	(21.0)
Male, 50	118	(17.9)
Female, 60	206	(31.3)
Male, 60	196	(29.8)
General education level, CASMIN	(*n* = 638)	
No completed/low	238	(37.3)
Mediate	285	(44.7)
High	115	(18.0)
Current employment status	(*n* = 649)	
Employed full-time	347	(53.5)
Employed part-time	191	(29.4)
Non-employed	111	(17.1)
Migration background	(*n* = 623)	
Yes	89	(14.3)
No	534	(85.7)
Prior stool test	(*n* = 648)	
Yes	511	(78.9)
No	137	(21.1)
Prior colonoscopy	(*n* = 646)	
Yes	362	(56.0)
No	284	(44.0)
Intention to participate in CRC screening	(*n* = 644)	
Yes, stool test	149	(23.1)
Yes, colonoscopy	249	(38.7)
Yes, but not yet decided on a procedure	98	(15.2)
No	148	(23.0)
BMI^b^ categories, WHO	(*n* = 656)	
Underweight/normal weight	220	(33.5)
Pre-obesity	232	(35.4)
Obesity class I-III	204	(31.1)
CRC history of first-degree relatives, parents	(*n* = 654)	
Yes	70	(10.7)
No	550	(84.1)
I don’t know	34	(5.2)

*BMI* body mass index, *CASMIN* comparative analysis of social mobility in industrial nations, *CRC* colorectal cancer, *WHO* World Health Organization

^a^Since 2025, women and men aged 50 have been eligible for a first colonoscopy as part of the organised colorectal cancer screening program. At the time the study was conducted, men aged 50 and women aged 55 were entitled to their first screening colonoscopy

^b^Calculated from self-reported height and weight

### Predicted uptake of screening tests in standard screening and screening extended by sigmoidoscopy

Overall, predicted uptakes based on the DCE preferences were highest for colonoscopy followed by stool test and sigmoidoscopy (Fig. 1). In extended screening, 22.5% (95%CI 16.2–28.9%) of women and 13.4% (95%CI 9.6–17.2%) of men would choose to undergo sigmoidoscopy if it was offered alternatively to stool test and colonoscopy. The extended screening could yield a slightly higher, but not significant total participation in CRC screening in both women (+ 5.8%; 95%CI -0.4-12.0%) and men (+ 3.1; 95%CI -3.4-9.6%). At the same time, participation of colonoscopy, stool test, and percentage of ‘no screening’ would decrease indicating a shift towards sigmoidoscopy.

In sensitivity analyses using effectiveness level values based on observational studies and ITS analyses from RCTs (see Additional File 1, Table 2; Figs. 1 and 2), the predicted uptake of sigmoidoscopy was slightly lower for both women and men compared with the main analysis. Estimates for colonoscopy, stool test, ‘no screening’, as well as total participation in CRC screening showed great variation.

### Estimated effects on CRC incidence and mortality

Estimates based on the test-specific effectiveness and the DCE-based uptake showed no relevant differences of the standard vs. the extended screening on CRC incidence and mortality (Fig. 2). The extended screening tends to have a slightly higher overall benefit with lower estimated CRC incidence and mortality indicating that shifts from stool tests and ‘no screening’ to sigmoidoscopy compensate for shifts away from colonoscopy (see Additional File 1, Tables 3 and 4).

### Comparison of DCE-based and intended uptake

**Fig. 3:**
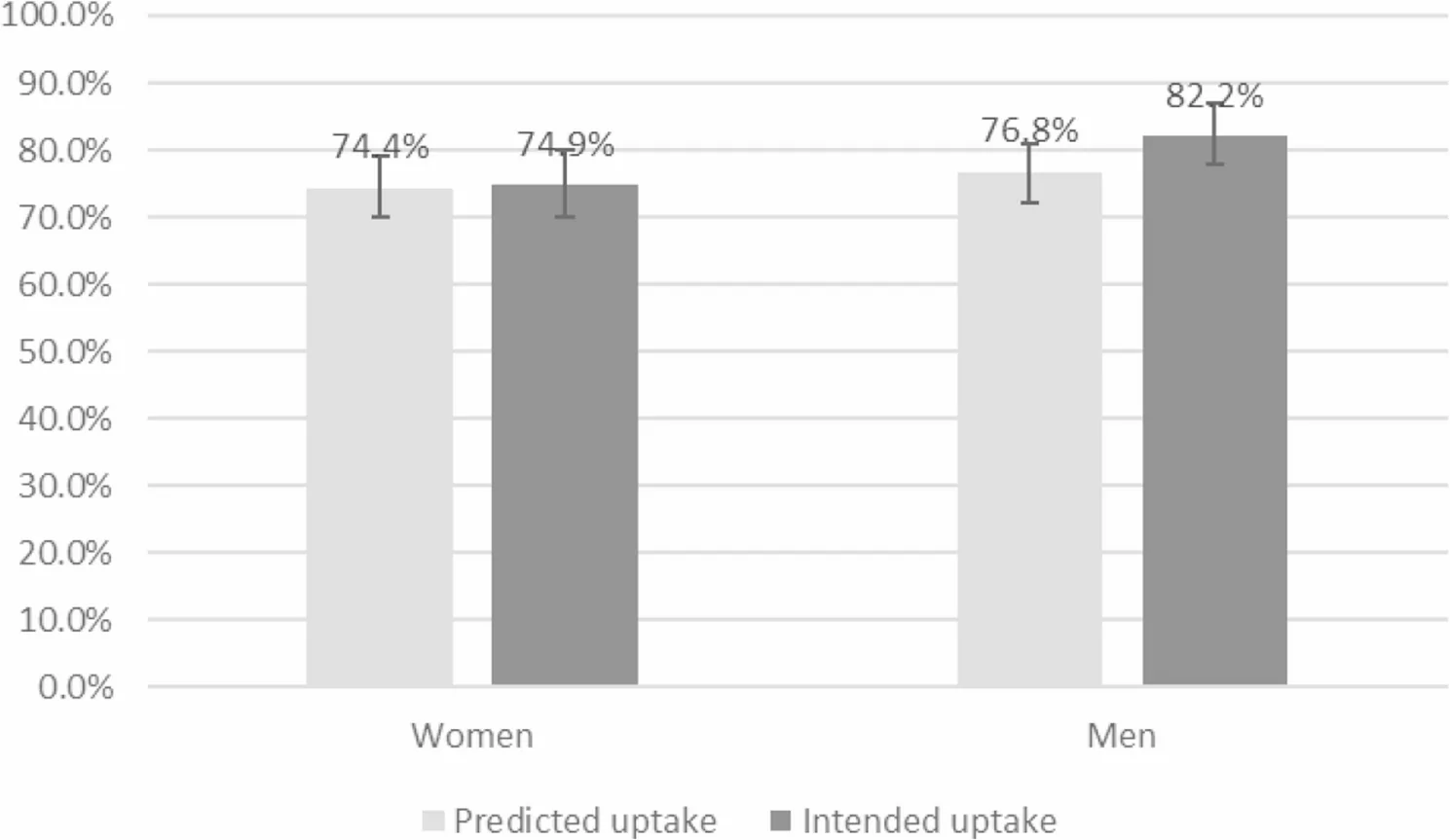
Comparison of preference-based and intended screening uptake at population level among women and men (Effectiveness level values were based on per-protocol (PP) analyses of randomised controlled trials (RCT))

The preference-predicted and the intended total uptake of CRC screening showed a good agreement, better in women than men (Fig. 3).

## Discussion

This DCE showed that a subgroup of participants would prefer sigmoidoscopy as an option for CRC screening. A greater proportion of women (22.5%; 95%CI 16.2–28.9%) than men (13.4%; 95%CI 9.6–17.2%) would decide themselves for the sigmoidoscopy, suggesting that preferences for this screening test depend at least on sex. The additional offer of sigmoidoscopy as an alternative screening test increases non-significantly overall participation in CRC screening, particularly among women. Compared with standard screening, the predicted uptake for colonoscopy and the stool test as well as for ‘no screening’ was slightly lower when sigmoidoscopy was offered as an additional option. However, these differences are unlikely to result in a lower overall effect on CRC-specific incidence and mortality compared to standard screening but might be compensated for, probably through both higher participation and shifts from stool test to sigmoidoscopy.

### Positive effect of screening extended by sigmoidoscopy: higher participation?

Higher participation in CRC screening is urgently demanded as projections indicate that by 2050 in an aging population, despite an already falling CRC incidence, the number of cases of CRC will substantially increase, which will put a strain on medical care [33, 36]. RCTs in Poland and the US have shown that participation is higher if both, colonoscopy and FIT, are offered instead a single test option [37, 38]. To our knowledge, evidence for incremental benefits of a third option for participation rates in CRC screening is lacking. More options may not necessarily increase participation due to decision overload or because preferences are not better satisfied by adding a third option [39]. Our estimated uptake based on preferences suggests that the additional offer of sigmoidoscopy may help increase participation by a few percentage points through an enhanced active choice strategy. This can be explained by the fact that each of the three tests incorporates different characteristics. Together they might allow a better fit to individual needs, which are highly heterogeneous in cancer screening as own results and a recent systematic review shows [15, 23, 40]. Sigmoidoscopy could be a relevant option for those who have problems with extensive laxative measures or those who do not want a day off from work due to sedation as with colonoscopy, and prefer the benefit of preventing CRC cancer, which is not as clear with stool tests. Future studies will show whether this applies in the real-world setting.

### Potential harms through shifts from colonoscopy to sigmoidoscopy

While extending CRC screening by sigmoidoscopy may increase overall participation, simultaneous shifts from colonoscopy to sigmoidoscopy could lower the effects on CRC incidence and mortality. In real healthcare, there might be only a very small shift to sigmoidoscopy from those who would have chosen colonoscopy in standard screening, as physicians usually recommend the gold standard colonoscopy as a previous survey in Germany showed [41]. Assuming the predicted shifts, preliminary analyses (see Fig. 2) showed no relevant differences in CRC incidence and mortality between the standard screening with stool test or colonoscopy vs. the extended screening with sigmoidoscopy based on the estimated uptakes. However, this addresses the potential impact on the populational level, while on the individual level the potential harms may include later detection of CRC with worse prognosis in those, who underwent sigmoidoscopy instead of colonoscopy or instead of stool test (i.e. in proximal CRC or precursors) [42]. Here, it is even more crucial that the eligible population receives evidence-based information including appropriate and understandable quantitative data to consider the benefits and harms of the available tests to support informed choices [15, 19].

### Barriers of implementing screening sigmoidoscopy

While our results support piloting the additional offer of the sigmoidoscopy, several barriers may threaten its successful implementation. In 2013, England initiated the implementation of a population-wide screening program with a 1-time sigmoidoscopy at the age of 55 (“bowel scope screening”) [43]. The program has already stopped for several reasons including less participation than expected (40 instead of 70%), and capacity problems, because in the meantime the introduction of the FIT with higher sensitivity than the former gFOBT resulted in significant increases in demand for colonoscopy following positive FITs’ [44, 45]. In the German healthcare system, different obstacles may occur. First, sigmoidoscopy is not very popular in Germany, where colonoscopy is highly established in outpatient healthcare for more than 20 years. In 2015, a survey among German gastroenterologists showed a low acceptance with nearly two-thirds rejecting sigmoidoscopy as an early detection method, mainly due to its incomplete bowel inspection compared to colonoscopy [41]. However, if subjects reject colonoscopy, 75% of physicians considered sigmoidoscopy to be an acceptable offer. Insurees may reject sigmoidoscopy, as well as colonoscopy, due to shame, embarrassment, anticipated pain, and preparation difficulties that the DCE did not include explicitly [46–48]. Pilot studies with regional implementation of sigmoidoscopy should explore different barriers in healthcare.

### How good do the DCE-based preferences estimate participation in CRC screening?

The preference-based predicted and the intended uptake are in good agreement validating that the collected DCE-based preferences reflect the individual’s intention with sufficient accuracy. This is in line with other findings in CRC screening and further health interventions [49, 50]. However, the predicted and intended uptake is much higher than the actual uptake in CRC screening in Germany, a phenomenon known as intention-behaviour gap [51]. This might be even pronounced in our study population, as AOK insurees compared to other insurees include on average more less educated subjects with less participation rates [52]. Overall, in this study, preferences are well in line with the intentions to participate, but should be used to predict relative differences in uptake rather than absolute uptake.

### Strengths and limitations

The main strength lies in assessing the population’s preferences using a proven experimental design and doing this for a procedure that isn’t offered in the present CRC screening program. Basically, the DCE is capable to depict the sigmoidoscopy well by using relevant procedure-specific attributes. Although the internal and external validity of the DCE can be considered sufficient in itself, the validity of the preferences estimated in the DCE is difficult to assess [53, 54]. Several assumptions had to be made, as the effectiveness of the various procedures, which were originally included as levels in the DCE, were not comparable due to different levels of evidence. Therefore, we adjusted the original levels of the attributes on risk reduction of CRC specific incidence and mortality to the newly introduced FIT, the recent results of the RCT on colonoscopy, different evidence levels, and analyses methods (PP, ITS) (see Additional File 1). Furthermore, the results of the RCT published in 2022 on the effectiveness of screening colonoscopy [9] have been controversially discussed, as incidence and mortality reduction were worse than expected, probably because the observation period was too short (underestimation of CRC mortality reduction), and prevalent CRC at baseline in the screening group were not excluded leading to an underestimated CRC risk reduction, that might have resulted in an underestimation of the preference-based predicted uptake of colonoscopy [55, 56]. Another potential limitation of this study is the relatively low response rate and the number of questionnaires finally included in the analysis, which may have resulted in selection bias and affected the representativeness and generalizability of the findings [23].

### Conclusions

A considerable absolute proportion of 10 to 29%, more women than men, would prefer CRC screening via sigmoidoscopy according to the DCE. Empowering the population to make decisions more in line with their preferences might slightly increase overall participation in CRC screening. These results should encourage policy makers and/or health providers to consider extending screening programs by sigmoidoscopy. The next steps include studies to examine the acceptance and feasibility of sigmoidoscopy for CRC screening in healthcare settings.

## Supplementary Information

Supplementary Material 1.

## Data Availability

The datasets generated and/or analysed during the current study are not publicly available due to legal restrictions but are available from the corresponding author on reasonable request.

## Abbreviations

CRC: Colorectal cancer
DCE: Discrete choice experiment
gFOBT: Guaiac-based faecal occult blood test
FIT: Faecal immunochemical test
ITS: Intention-to-screen
PP: Per-protocol
RCT: Randomised controlled trial
SIGMO: Sigmoidoscopy as an evidence based colorectal cancer screening test – a possible option? (Acronym)
