# Supplementary material for: The role of sigmoidoscopy in colorectal cancer screening – public preferences and predicted uptake
Source: BMC Public Health. 2026 Nov 13;26:3377. doi: 10.1186/s12889-026-30227-w (PMC13760816; doi:10.1186/s12889-026-30227-w)
Supplement: Supplementary file 1 [file 12889_2026_30227_MOESM1_ESM.pdf]

Additional file 1

Table 1. Definition of screening tests in standard CRC screening (stool test or colonoscopy) and standard screening extended by sigmoidoscopy for the analysis of the indirect utility and probability of uptake for screening tests with effectiveness level values based on observational studies, per-protocol (PP) analyses and intention-to-screen (ITS) analyses of randomised controlled trials (RCT).

Study design	Screening test	Attributes and levels							
		Incidence	Reference	Mortality	Reference	Complications	Preparation	Need for transportation	Follow-up
**Observational studies**	Colonoscopy	3.1/1000[a]	1	0.9/1000[a]	1	2/1000	Laxatives	Almost always	0/1000
	Sigmoidoscopy	5.1/1000[a]	2	1.5/1000[a]	2	1/1000	Enema	Occasionally	440/1000
	FIT	9.0/1000[b]	2,3	1.9/1000[b]	2,3	0/1000	None	Never	340/1000
	No screening	10.0/1000[c]	4	2.9/1000[c]	4	0/1000	None	Never	0/1000
**RCTs (PP-Analyses)**	Colonoscopy	6.9/1000[a]	1	1.5/1000[a]	1	2/1000	Laxatives	Almost always	0/1000
	Sigmoidoscopy	7.2/1000[a]	2	1.6/1000[a]	2	1/1000	Enema	Occasionally	440/1000
	FIT	9.4/1000[b]	2,3	2.0/1000[b]	2,3	0/1000	None	Never	340/1000
	No screening	10.0/1000[c]	4	2.9/1000[c]	4	0/1000	None	Never	0/1000
**RCTs (ITS-Analyses)**	Colonoscopy	8.2/1000[a]	1	2.6/1000[a]	1	2/1000	Laxatives	Almost always	0/1000
	Sigmoidoscopy	8.2/1000[a]	2	2.1/1000[a]	2	1/1000	Enema	Occasionally	440/1000
	FIT	9.6/1000[b]	2,3	2.3/1000[b]	2,3	0/1000	None	Never	340/1000
	No screening	10.0/1000[c]	4	2.9/1000[c]	4	0/1000	None	Never	0/1000

CRC=colorectal cancer; FIT=immunochemical faecal occult blood test

[a]Absolute risks were calculated by applying relative risks from observational studies, per-protocol, and intention-to-screen analyses of randomized controlled trials to the average risk of developing and dying from colorectal cancer among 55-year-old women and men in the next 10 years in Germany.

[b]Corresponds to the ratio in effectiveness between FIT and sigmoidoscopy from observational studies.

[c]Corresponds to the average risk of developing and dying from colorectal cancer among 55-year-old women and men in the next 10 years in Germany.

References:

1. Bretthauer M, Løberg M, Wieszczy P, et al. Effect of Colonoscopy Screening on Risks of Colorectal Cancer and Related Death. New England Journal of Medicine. 2022;387(17):1547-1556. doi:10.1056/nejmoa2208375.

2. Brenner H, Stock C, Hoffmeister M. Effect of screening sigmoidoscopy and screening colonoscopy on colorectal cancer incidence and mortality: systematic review and meta-analysis of randomised controlled trials and observational studies. BMJ. 2014;348(apr09 1):g2467-g2467. doi:10.1136/bmj.g2467.
3. Rossi PG, Vicentini M, Sacchettini C, et al. Impact of Screening Program on Incidence of Colorectal Cancer: A Cohort Study in Italy. Official journal of the American College of Gastroenterology | ACG. 2015;110(9):1359-1366. doi:10.1038/ajg.2015.240.
4. Institut für Qualität und Wirtschaftlichkeit im Gesundheitswesen. IQWiG-Berichte – Nr. 451. Einladungsschreiben und Entscheidungshilfen zum Darmkrebs-Screening. Accessed 13.06.2025, https://www.iqwig.de/download/p15-01_abschlussbericht_einladungsschreiben-und-entscheidungshilfe-zum-darmkrebs-screening.pdf

Table 2. Indirect utility (V) and probability of uptake for screening tests (P) in standard colorectal cancer screening (stool test or colonoscopy) and standard screening extended by sigmoidoscopy (+ sigmoidoscopy) among women and men with effectiveness level values based on observational studies, per-protocol (PP), and intention-to-screen (ITS) analyses of randomised controlled trials (RCT).

Screening test	V	95% CI		Stool test/colonoscopy			+ sigmoidoscopy		
				P	95% CI		P	95% CI	
**Effectiveness level values based on observational studies**									
**Women**									
Colonoscopy	-2.702	-3.149	-2.255	0.749	0.633	0.865	0.619	0.501	0.737
Sigmoidoscopy	-3.971	-4.558	-3.384				0.174	0.119	0.229
FIT	-4.305	-4.981	-3.629	0.151	0.083	0.218	0.125	0.072	0.177
No screening	-4.717	-5.479	-3.955	0.100	0.048	0.151	0.083	0.041	0.124
Total uptake (TU)[1]				0.900	0.849	0.952	0.917	0.876	0.959
Gain in TU[2]							0.017	-0.027	0.061
**Men**									
Colonoscopy	-1.740	-2.062	-1.418	0.747	0.641	0.853	0.671	0.562	0.779
Sigmoidoscopy	-3.619	-4.147	-3.090				0.102	0.069	0.136
FIT	-3.370	-3.951	-2.788	0.146	0.087	0.206	0.131	0.081	0.182
No screening	-3.689	-4.349	-3.029	0.106	0.057	0.156	0.096	0.052	0.139
Total uptake (TU) [1]				0.894	0.844	0.943	0.904	0.861	0.948
Gain in TU[2]							0.010	-0.040	0.060
**Effectiveness level values based on RCTs (PP-analyses)**									
**Women**									
Colonoscopy	-4.210	-4.824	-3.597	0.425	0.297	0.554	0.329	0.231	0.428
Sigmoidoscopy	-4.589	-5.251	-3.927				0.225	0.162	0.289
FIT	-4.498	-5.200	-3.797	0.319	0.245	0.392	0.247	0.187	0.307
No screening	-4.717	-5.479	-3.955	0.256	0.188	0.324	0.198	0.138	0.259
Total uptake (TU) [1]				0.744	0.676	0.812	0.802	0.741	0.862
Gain in TU[2]							0.058	-0.010	0.126
**Men**									
Colonoscopy	-2.951	-3.400	-2.503	0.490	0.374	0.606	0.424	0.323	0.526
Sigmoidoscopy	-4.105	-4.694	-3.517				0.134	0.096	0.172
FIT	-3.526	-4.129	-2.923	0.276	0.212	0.340	0.239	0.184	0.294
No screening	-3.689	-4.349	-3.029	0.234	0.172	0.297	0.203	0.146	0.260
Total uptake (TU) [1]				0.766	0.703	0.828	0.797	0.740	0.854
Gain in TU[2]							0.031	-0.028	0.090
**Effectiveness level values based on RCTs (ITS - analyses)**									
**Women**									
Colonoscopy	-5.559	-6.337	-4.781	0.185	0.112	0.259	0.150	0.094	0.205
Sigmoidoscopy	-5.304	-6.055	-4.553				0.193	0.130	0.256

FIT	-4.827	-5.573	-4.082	0.385	0.332	0.437	0.311	0.264	0.358
No screening	-4.717	-5.479	-3.955	0.430	0.375	0.484	0.347	0.282	0.412
Total uptake (TU) [1]				0.570	0.516	0.625	0.653	0.588	0.718
Gain in TU[2]							0.083	0.010	0.156
**Men**									
Colonoscopy	-4.069	-4.660	-3.479	0.265	0.186	0.344	0.232	0.166	0.299
Sigmoidoscopy	-4.693	-5.356	-4.030				0.124	0.086	0.163
FIT	-3.801	-4.442	-3.160	0.347	0.296	0.398	0.304	0.258	0.349
No screening	-3.689	-4.349	-3.029	0.388	0.336	0.439	0.340	0.284	0.395
Total uptake (TU) [1]				0.612	0.561	0.664	0.660	0.605	0.716
Gain in TU[2]							0.048	-0.025	0.121

V=indirect utility; P=probability of uptake; CI=confidence interval; FIT=immunochemical fecal occult blood test; RCTs=randomized controlled trials; PP=per-protocol; ITS=intention-to-screen

[1]Calculated as complementary to 'no screening'

[2]Difference: total uptake (+ sigmoidoscopy) - total participation (stool test/colonoscopy)

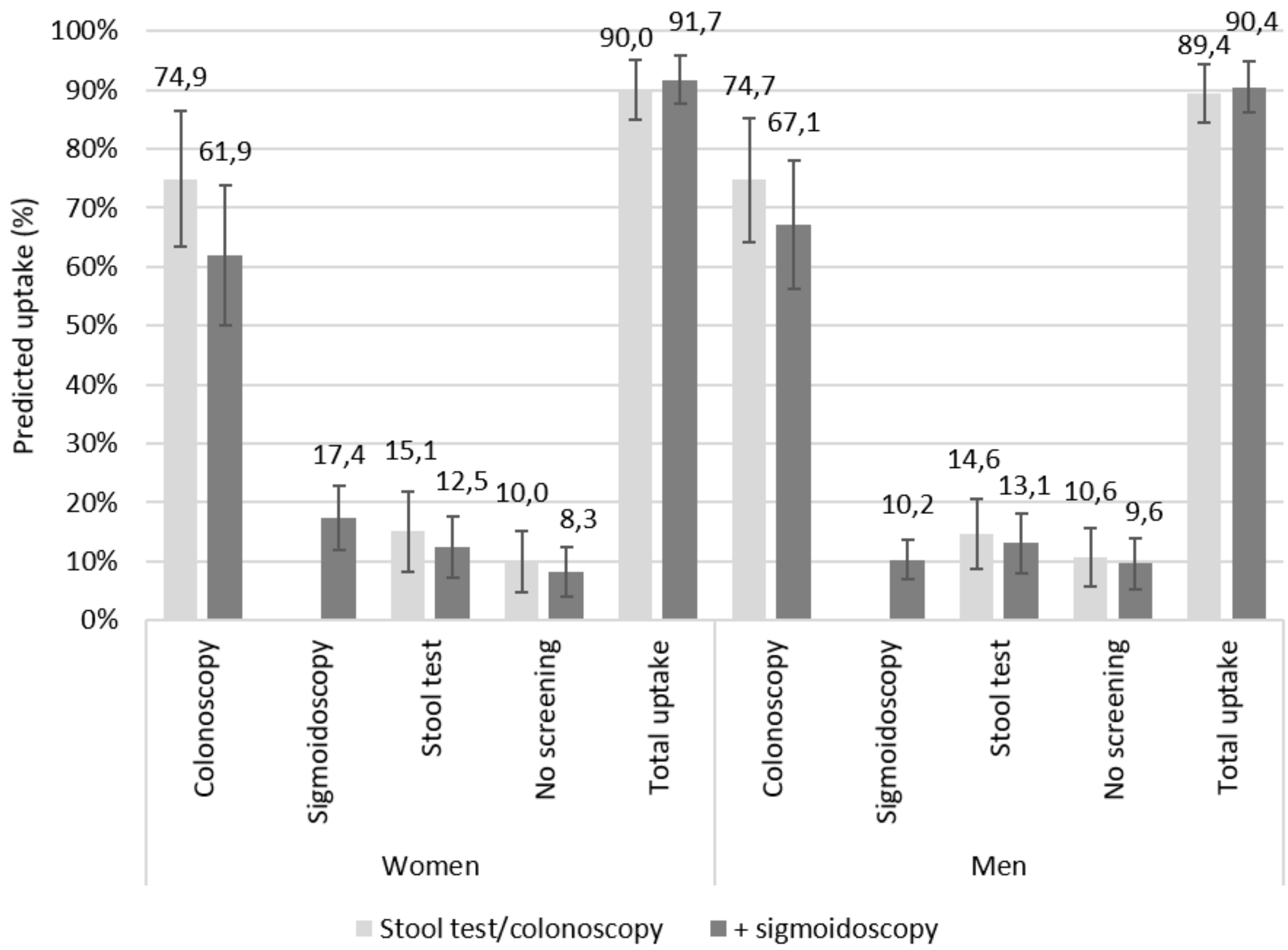

Figure 1. Predicted uptake (%) for colorectal cancer screening tests in standard screening (stool test /colonoscopy) and screening extended by sigmoidoscopy (+ sigmoidoscopy) among women and men with effectiveness level values based on observational studies.

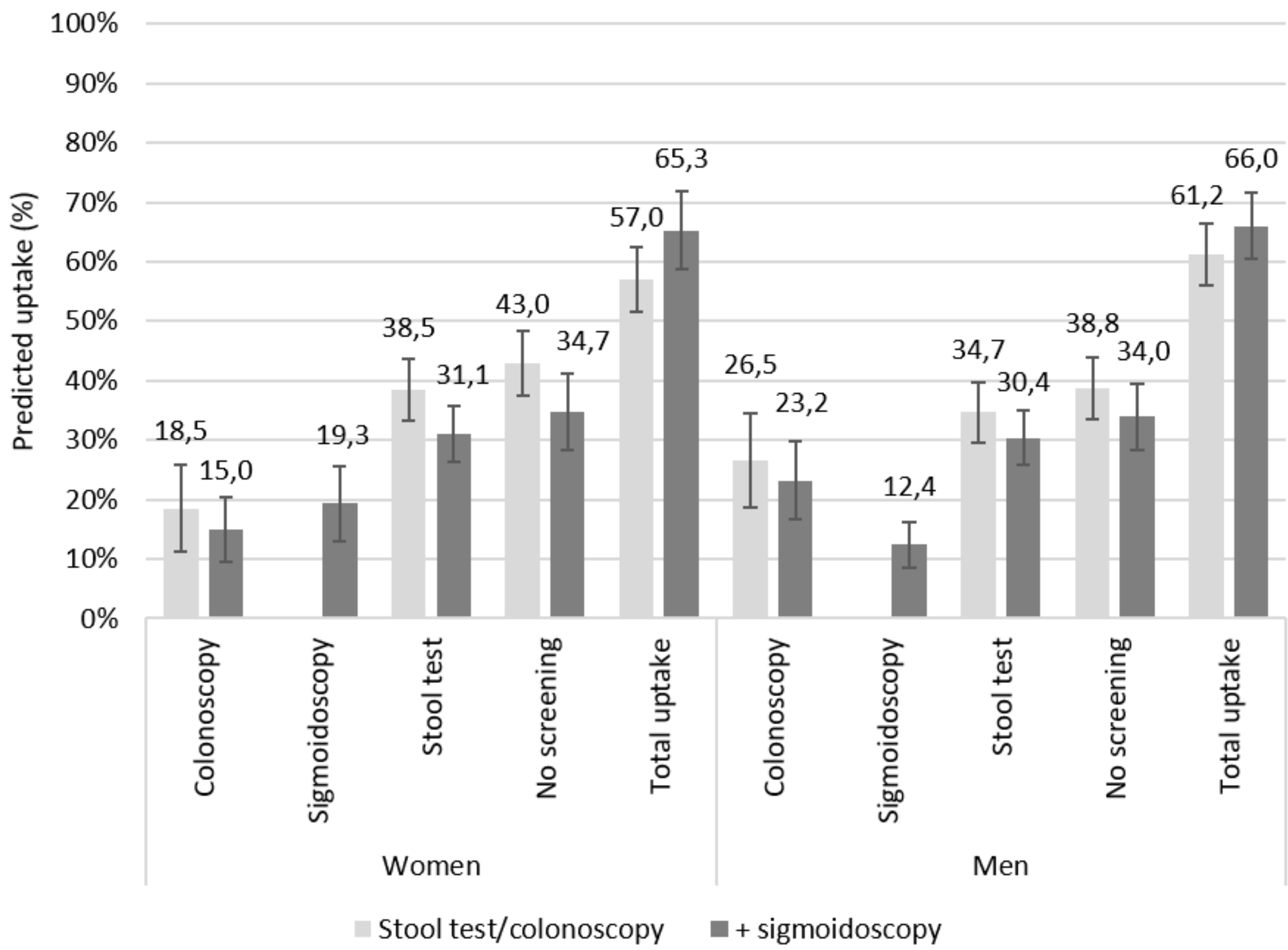

Figure 2. Predicted uptake (%) for colorectal cancer screening tests in standard screening (stool test /colonoscopy) and screening extended by sigmoidoscopy (+ sigmoidoscopy) among women and men with effectiveness level values based on intention-to-screen (ITS) analyses of randomized controlled trials (RCTs).

Table 3. Effect of standard CRC screening (stool test or colonoscopy) compared with screening extended by sigmoidoscopy (+ sigmoidoscopy) on cancer-specific incidence and mortality with effectiveness level values based on observational studies, per-protocol (PP), and intention-to-screen (ITS) analyses of randomised controlled trials (RCT) in **women.**

Screening test	Effectiveness level values (E)		Standard screening (stool test, colonoscopy) (s1)			Extended screening (s2)			Difference between extended and standard screening (/1,000)[2]
	/1,000	/100	P	P (/1,000)	Develop/die from CRC (/1,000)[1]	P	P (/1000)	Develop/die from CRC (/1,000)[1]	
**Effectiveness level values based on observational studies**									
**Incidence**									
Fecal testing	9.0	0.90	0.151	151	1.36	0.125	125	1.13	
Colonoscopy	3.1	0.31	0.749	749	2.32	0.619	619	1.92	
No screening	10.0	1.00	0.100	100	1.00	0.083	83	0.83	
Sigmoidoscopy	5.1	0.51				0.174	174	0.89	
Sum			0.900		4.68	0.918		4.76	-0.080
**Mortality**									
Fecal testing	1.9	0.19	0.151	151	0.29	0.125	125	0.24	
Colonoscopy	0.9	0.09	0.749	749	0.67	0.619	619	0.56	
No screening	2.9	0.29	0.100	100	0.29	0.083	83	0.24	
Sigmoidoscopy	1.5	0.15				0.174	174	0.26	
Sum			0.900		1.25	0.918		1.30	-0.045
**Effectiveness level values based on RCT (PP analyses)**									
**Incidence**									
Fecal testing	9.4	0.94	0.319	319	3.00	0.247	247	2.32	
Colonoscopy	6.9	0.69	0.425	425	2.93	0.329	329	2.27	
No screening	10.0	1.00	0.256	256	2.56	0.198	198	1.98	
Sigmoidoscopy	7.2	0.72				0.225	225	1.62	
Sum			0.744		8.49	0.801		8.19	0.299
**Mortality**									
Fecal testing	2.0	0.20	0.319	319	0.63	0.247	247	0.49	
Colonoscopy	1.5	0.15	0.425	425	0.64	0.329	329	0.49	
No screening	2.9	0.29	0.256	256	0.74	0.198	198	0.57	
Sigmoidoscopy	1.6	0.16				0.225	225	0.36	

Screening test	Effectiveness level values (E)		Standard screening (stool test, colonoscopy) (s1)			Extended screening (s2)			Difference between extended and standard screening (/1,000)[2]
	/1,000	/100	P	P (/1,000)	Develop/die from CRC (/1,000)[1]	P	P (/1000)	Develop/die from CRC (/1,000)[1]	
Sum			0.744		2.02	0.801		1.92	0.096
**Effectiveness level values based on RCT (ITS analyses)**									
**Incidence**									
Fecal testing	9.6	0.96	0.385	385	3.70	0.311	311	2.99	
Colonoscopy	8.2	0.82	0.185	185	1.52	0.150	150	1.23	
No screening	10.0	1.00	0.430	430	4.30	0.347	347	3.47	
Sigmoidoscopy	8.2	0.82				0.193	193	1.58	
Sum			0.570		9.51	0.654		9.27	0.245
**Mortality**									
Fecal testing	2.3	0.23	0.385	385	0.89	0.311	311	0.72	
Colonoscopy	2.6	0.26	0.185	185	0.48	0.150	150	0.39	
No screening	2.9	0.29	0.430	430	1.25	0.347	347	1.01	
Sigmoidoscopy	2.1	0.21				0.193	193	0.41	
Sum			0.570		2.61	0.654		2.52	0.097

CRC=colorectal cancer; P= probability of uptake; 1= annually
2=absolute risk reduction (CRC incidence/mortality)= Σ (Es2 x Ps2) - Σ (Es1 x Ps1);

Table 4. Effect of standard CRC screening (stool test or colonoscopy) compared with screening extended by sigmoidoscopy (+ sigmoidoscopy) on cancer-specific incidence and mortality with effectiveness level values based on observational studies, per-protocol (PP), and intention-to-screen (ITS) analyses of randomised controlled trials (RCT) in **men.**

Screening test	Effectiveness level values		Standard screening (stool test, colonoscopy) (s1)			Extended screening (s2)			Difference between extended and standard screening (/1,000)[2]
	/1,000	/100	P	P (/1,000)	Develop/die from CRC (/1,000)[1]	P	P (/1,000)	Develop/die from CRC (/1,000)[1]	
**Effectiveness level values based on observational studies**									
**Incidence**									
Fecal testing	9.0	0.90	0.146	146	1.31	0.131	131	1.18	
Colonoscopy	3.1	0.31	0.747	747	2.32	0.671	671	2.08	
No screening	10.0	1.00	0.106	106	1.06	0.096	96	0.96	
Sigmoidoscopy	5.1	0.51				0.102	102	0.52	
Sum			0.893		4.69	0.904		4.74	-0.050
**Mortality**									
Fecal testing	1.9	0.19	0.146	146	0.28	0.131	131	0.25	
Colonoscopy	0.9	0.09	0.747	747	0.67	0.671	671	0.60	
No screening	2.9	0.29	0.106	106	0.31	0.096	96	0.28	
Sigmoidoscopy	1.5	0.15				0.102	102	0.15	
Sum			0.893		1.26	0.904		1.28	-0.027
**Effectiveness level values based on RCT (PP analyses)**									
**Incidence**									
Fecal testing	9.4	0.94	0.276	276	2.60	0.239	239	2.25	
Colonoscopy	6.9	0.69	0.490	490	3.38	0.424	424	2.93	
No screening	10.0	1.00	0.230	230	2.30	0.203	203	2.03	
Sigmoidoscopy	7.2	0.72				0.134	134	0.96	
Sum			0.766		8.28	0.797		8.17	0.108
**Mortality**									
Fecal testing	2.0	0.20	0.276	276	0.55	0.239	239	0.48	
Colonoscopy	1.5	0.15	0.490	490	0.73	0.424	424	0.64	
No screening	2.9	0.29	0.230	230	0.67	0.203	203	0.59	
Sigmoidoscopy	1.6	0.16				0.134	134	0.21	

Screening test	Effectiveness level values		Standard screening (stool test, colonoscopy) (s1)			Extended screening (s2)			Difference between extended and standard screening (/1,000)[2]
	/1,000	/100	P	P (/1,000)	Develop/die from CRC (/1,000)[1]	P	P (/1,000)	Develop/die from CRC (/1,000)[1]	
Sum			0.766		1.95	0.797		1.92	0.037
**Effectiveness level values based on RCT (ITS analyses)**									
**Incidence**									
Fecal testing	9.6	0.96	0.347	347	3.33	0.304	304	2.92	
Colonoscopy	8.2	0.82	0.265	265	2.17	0.232	232	1.90	
No screening	10.0	1.00	0.388	388	3.88	0.340	340	3.40	
Sigmoidoscopy	8.2	0.82				0.124	124	1.02	
Sum			0.612		9.38	0.66		9.24	0.147
**Mortality**									
Fecal testing	2.3	0.23	0.347	347	0.80	0.304	304	0.70	
Colonoscopy	2.6	0.26	0.265	265	0.69	0.232	232	0.60	
No screening	2.9	0.29	0.388	388	1.13	0.340	340	0.99	
Sigmoidoscopy	2.1	0.21				0.124	124	0.26	
Sum			0.612		2.61	0.660		2.55	0.063

CRC=colorectal cancer; P= probability of uptake; 1= annually
2=absolute risk reduction (CRC incidence/mortality)= Σ (Es2 x Ps2) - Σ (Es1 x Ps1);
